# Bibliometric and visual analysis of diabetic keratopathy research: trends, collaborations, and future directions

**DOI:** 10.3389/fmed.2024.1468402

**Published:** 2024-10-18

**Authors:** Wang Zhenyu, Gao Jing, Wu Tianhong

**Affiliations:** Department of Ophthalmology, The First Affiliated Hospital of Soochow University, Suzhou, Jiangsu, China

**Keywords:** diabetes, cornea, ocular surface, confocal microscopy, peripheral neuropathy, bibliometrics

## Abstract

**Purpose:**

Diabetic keratopathy has gained increasing attention due to advancements in diagnostic and therapeutic techniques a. This article presents a visual and bibliometric analysis to illustrate the knowledge network, research hotspots, trends, and potential future directions in this field.

**Methods:**

We retrieved articles published since 2000 from the Web of Science and analyzed the authors, institutions, countries, keywords, citations, and co-citations of these articles with VOSviewer and CiteSpace.

**Results:**

A total of 706 highly relevant articles were identified, with the United States, China and England as major contributors; the University of Manchester, Queensland University of Technology and Weill Cornell Medical−Qatar as key institutions; and Malik Rayaz, Efron Nathan and Ferdousi Maryam as prominent authors. High-citation articles have focused mainly on corneal confocal microscopy and diabetic peripheral neuropathy. Keywords form two clusters: one around complications, diabetes and cornea sensitivity, and another around corneal confocal microscopy and peripheral neuropathy.

**Conclusion:**

The identification of diabetic peripheral neuropathy via corneal confocal microscopy has been a major focus of research in this field, but the mechanisms underlying diabetic corneal neuropathy still require further investigation and breakthroughs.

## 1 Introduction

The increasing global prevalence of diabetes has made it a significant health issue and socioeconomic burden, with more than 600 million individuals projected to be affected by diabetes by 2040 (1). Diabetes-related eye diseases, which manifest early in the course of diabetes, are a leading cause of blindness in both developed and developing countries (2). Initially, diabetic retinopathy and cataracts were the primary focus. However, with advancements in diagnostic techniques, diabetic keratopathy (DK) has garnered increasing attention from clinicians and researchers (3, 4).

DK encompasses a range of corneal alterations caused by diabetes, varying in form and severity. Clinically, DK manifests as superficial punctate keratopathy, delayed epithelial healing, persistent epithelial defects or recurrent erosions, nerve loss, decreased corneal sensitivity, tear film changes, ulcers, and opacities (5, 6). These changes impair patients’ quality of life and vision to varying degrees and are often underestimated in clinical settings, with conventional treatments yielding limited efficacy (7). Understanding the pathogenesis and key factors of DK is crucial for effective treatment.

Bibliometrics, involving the quantitative analysis of scientific publications using mathematical and statistical methods, focuses on citations, publications, co-authorship, journals, keywords, and research trends. Analyzing a large volume of literature provides insights into the structure, development, and dynamics of a field, guiding future research and development (8, 9).

In this study, we employed bibliometric methods to analyze literature on diabetic keratopathy published since the new millennium and presented our findings with visualization software. Our analysis addresses four questions: 1. What are the research trends in diabetic keratopathy? 2. Which countries, institutions, and authors are influential in this field? 3. What are the collaboration networks among leading institutions and authors? 4. What are the current research hotspots, keywords, and potential future directions in this field?

## 2 Materials and methods

### 2.1 Literature retrieval

We conducted a literature search using the Web of Science, focusing on the Web of Science Core Collection and the SCIE citation database from 1900 to the present. The search formula was “TS = [(cornea or corneal or keratopathy) and (diabetes or diabetic)] not (retina or retinal)”, with publication dates limited from 2000 to the present (up to May 20, 2024). Document types were restricted to articles or reviews, and the language was limited to English. Two authors independently screened the initial results to exclude studies and articles not primarily focused on ophthalmology or corneal disease. Discrepancies were resolved through discussion. The final selected literature records, which included all the citation information, were exported from the Web of Science.

### 2.2 Bibliometric analysis

We used VOSviewer and CiteSpace to perform bibliometric analysis and visualization of the exported literature records. The countries, institutions, authors, and keywords of the articles were clustered and visualized using VOSviewer. Journal impact factors and JCR rankings (2022) were obtained from the Web of Science. Time zone view of keywords and burst word analysis was conducted and presented using CiteSpace.

## 3 Results

### 3.1 General Information

**FIGURE 1:**
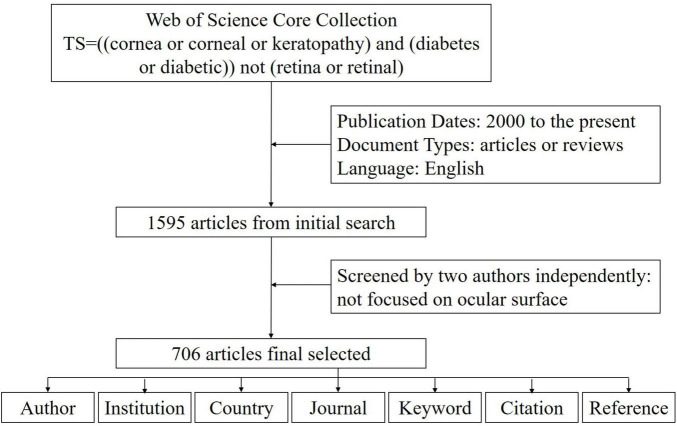
Data screening flow chart and steps of bibliometric analysis. The literature records were exported from the Web of Science.

**FIGURE 2:**
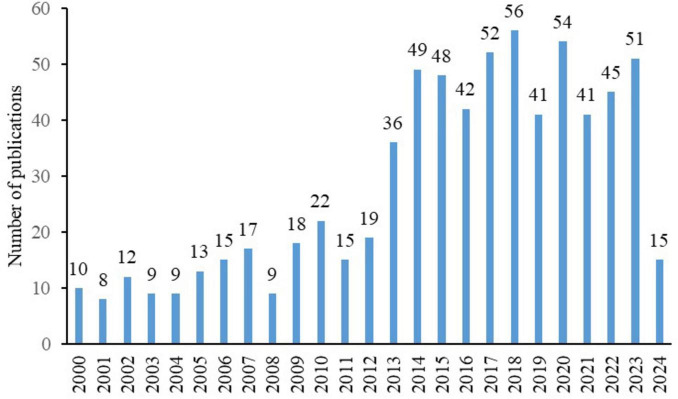
Annual publication volume of diabetic keratopathy research (2000–2024), highlighting the significant increase after 2013.

Our initial search yielded 1595 articles, 706 of which met the inclusion criteria after independent screening by two authors (Figure 1). These articles originated from 57 countries, 731 institutions, and 2670 authors and were published in 172 journals, with 13093 references from 2613 journals. The annual publication volume in this field showed a fluctuating upward trend, with 2013 being a significant turning point. Before 2013, the annual publication volume was between 10 and 20, but after 2013, it surged to between 40 and 50 (Figure 2).

### 3.2 Countries

**FIGURE 3:**
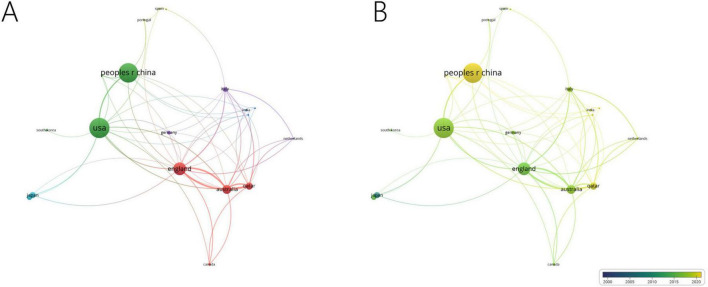
Collaboration network of leading countries. **(A)** The cooperative network visualization map of countries working on DK. Node size indicates the number of publications, the thickness of the link positively correlates with cooperation strength, and different colors represent different cooperative clusters. **(B)** The cooperative network overlay visualization map of countries working on DK. Blue represents the early research phase, and yellow represents the recent period.

Among the 57 countries publishing in this research field, 29 had more than five articles. Table 1 lists the top ten countries by publication volume, with the United States (average citations 15.65), China (average citations 13.79) and England (average citations 38.12) leading. Analysis of collaboration among countries with more than eight publications revealed two major cooperation clusters: one including the United States and China, and another including England, Australia and Qatar (Figures 3a, b).

**TABLE 1:** The top 10 prolific countries in the field of diabetic keratopathy.

Country	Publications	Percentage	Citations	Average citations
United States	130	18.4%	2034	15.65
China	126	17.8%	1737	13.79
England	76	10.8%	2897	38.12
Australia	47	6.7%	1839	39.13
Qatar	39	5.5%	879	22.54
Japan	34	4.8%	1109	32.62
Turkey	27	3.8%	447	16.56
Italy	26	3.7%	419	16.12
Germany	25	3.5%	617	24.68
India	13	1.8%	136	10.46

### 3.3 Institutions

**FIGURE 4:**
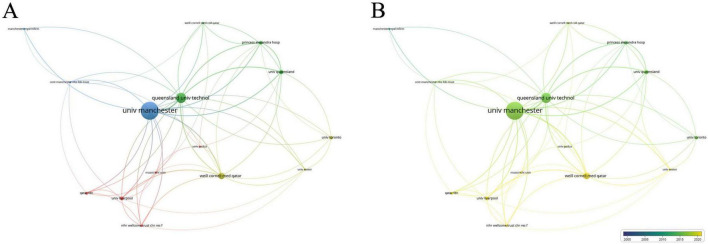
Collaboration network of leading institutions. **(A)** The cooperative network visualization map of institutions working on DK. Node size indicates the number of publications, the thickness of the link positively correlates with cooperation strength, and different colors represent different cooperative clusters. **(B)** The cooperative network overlay visualization map of institutions working on DK. Blue represents the early research phase, and yellow represents the recent period.

Of the 731 institutions publishing in this area, 64 had more than five publications. Table 2 lists the top ten institutions, with the University of Manchester far leading (71 publications, average citations 61.62), followed by Queensland University of Technology (40 publications, average citations 62.58) and Weill Cornell Medicine – Qatar (26 publications, average citations 29.62). Analysis of collaboration among institutions with over seven publications revealed two major collaboration centers: the University of Manchester and Weill Cornell Medicine - Qatar (Figures 4a, b).

**TABLE 2:** The top 10 prolific institution in the field of diabetic keratopathy.

Institution	Country	Publications	Percentage	Citations	Average citations
University of Manchester	England	71	10.1%	4375	61.62
Queensland University of Technology	Australia	40	5.7%	2503	62.58
Weill Cornell Medicine−Qatar	Qatar	26	3.7%	770	29.62
Cedars-Sinai Medical Center	United States	17	2.4%	716	42.12
University of Queensland	Australia	17	2.4%	807	47.47
Shandong Academy of Medical Sciences	China	16	2.3%	715	44.69
University of California, Los Angeles	United States	16	2.3%	652	40.75
Wayne State University	United States	16	2.3%	852	53.25
University of Liverpool	England	15	2.1%	486	32.40
Pennsylvania State University	United States	14	2.0%	388	27.71

### 3.4 Authors

**FIGURE 5:**
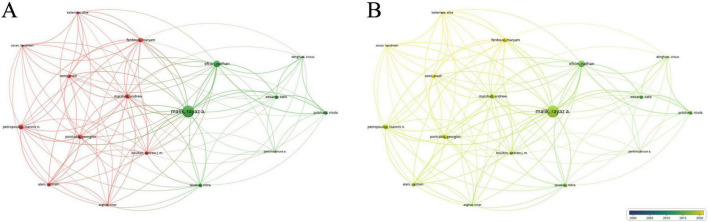
Collaboration network of leading authors. **(A)** The cooperative network visualization map of authors working on DK. Node size indicates the number of publications, and the thickness of the link positively correlates with cooperation strength, and different colors represent different cooperative clusters. **(B)** The cooperative network overlay visualization map of authors working on DK. Blue represents the early research phase, and yellow represents the recent period.

Among the 2670 authors who have published research results in this field, 91 authors have published more than 5 papers. Table 3 lists the top ten authors, with Malik Rayaz (60 publications, average citations 65.32), Efron Nathan (42 publications, average citations 74.6) and Ferdousi Maryam (25 publications, average citations 38.84) leading. Analysis of collaboration among authors with over 12 publications revealed two core networks: one led by Malik Rayaz and Efron Nathan, and another including Ponirakis Georgios, Ferdousi Maryam, Marshail Andrew, Petropoulos Ioannis N. and so on (Figures 5a, b).

**TABLE 3:** The top 10 prolific authors in the field of diabetic keratopathy.

Author	Country	Publications	Percentage	Citations	Average citations
Malik Rayaz	Qatar	60	8.5%	3919	65.32
Efron Nathan	Australia	42	5.9%	3133	74.6
Ferdousi Maryam	England	25	3.5%	971	38.84
Ponirakis Georgios	Qatar	24	3.4%	1334	55.58
Tavakoli Mitra	England	23	3.3%	2247	97.7
Petropoulos Ioannis N. N.	Qatar	22	3.1%	954	43.36
Ljubimov Alexander	United States	21	3.0%	1062	50.57
Edwards Katie	Australia	21	3.0%	931	44.33
Boulton Andrew J. M.	England	20	2.8%	2277	113.85
Azmi Shazli	England	20	2.8%	767	38.35

### 3.5 Journals

A total of 172 journals published research in this field, with 36 having more than five publications. Table 4 lists the top ten journals, primarily from the ophthalmology and diabetes fields. The leading journals included Investigative Ophthalmology Visual Science (impact factor 4.4, 75 publications, average citations 49.59), JCR Ophthalmology Category Q1, Cornea (impact factor 2.8, 53 publications, average citations 30.56), JCR Ophthalmology Category Q2, and Experimental Eye Research (impact factor 3.4, 30 publications, average citations 21.7), JCR Ophthalmology Category Q2.

**TABLE 4:** The top 10 Journals that published articles in diabetic keratopathy.

Journal	Impact factor (2022)	Publications	Percentage	Citations	Average citations
Investigative Ophthalmology Visual Science	4.4	75	10.6%	3719	49.59
Cornea	2.8	53	7.5%	1620	30.56
Experimental Eye Research	3.4	30	4.2%	651	21.7
Diabetes	7.7	23	3.3%	1438	62.5
Journal of Diabetes Investigation	3.2	18	2.5%	311	17.29
Current Eye Research	2.0	17	2.4%	306	18
Diabetes Care	16.2	17	2.4%	1913	112.54
BMC Ophthalmology	2.0	14	2.0%	95	6.82
International Journal of Ophthalmology	1.4	13	1.8%	207	15.9
Diabetologia	1.83	12	1.7%	916	76.33

### 3.6 Highly cited articles

**FIGURE 6:**
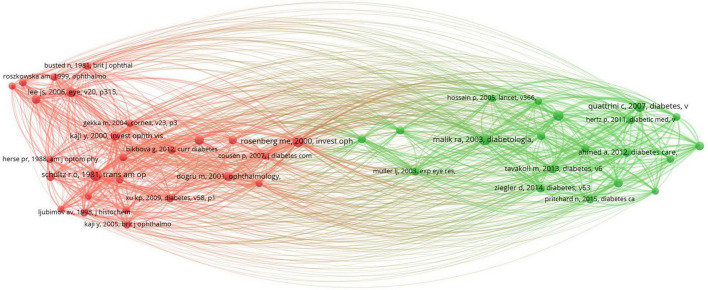
The visualization network of highly co-citated references. Node size indicates the frequency of co-citations, and different colors represent different co-citation clusters.

Studying highly cited articles in this field helps us understand the research hotspots in the area. Table 5 lists the top ten most cited articles, predominantly focusing on corneal confocal microscopy and diabetic peripheral neuropathy. Table 6 lists the top ten co-cited articles, highlighting keywords like corneal confocal microscopy, diabetic complications, ocular surface structure and so on. Clustering of references with over 40 co-citations revealed two major clusters (Figure 6). The red cluster is mainly about diabetic complications, cornea structure and so on, while the green cluster is focusing on corneal confocal microscopy.

**TABLE 5:** The top 10 most cited articles related to diabetic keratopathy.

Title	Journal	First author	Year	Citations
Corneal confocal microscopy: a non-invasive surrogate of nerve fibre damage and repair in diabetic patients	Diabetologia	Malik, RA	2003	366
Corneal structure and sensitivity in type 1 diabetes mellitus	Investigative Ophthalmology Visual Science	Rosenberg, ME	2000	319
Corneal Confocal Microscopy A novel noninvasive test to diagnose and stratify the severity of human diabetic neuropathy	Diabetes Care	Tavakoli, M	2010	266
Early Detection of Nerve Fiber Loss by Corneal Confocal Microscopy and Skin Biopsy in Recently Diagnosed Type 2 Diabetes	Diabetes	Ziegler, D	2014	229
Tear function and ocular surface changes in noninsulin-dependent diabetes mellitus	Ophthalmology	Dogru, M	2001	226
Automatic analysis of diabetic peripheral neuropathy using multi-scale quantitative morphology of nerve fibres in corneal confocal microscopy imaging	Medical Image Analysis	Dabbah, MA	2011	220
Corneal nerve tortuosity in diabetic patients with neuropathy	Investigative Ophthalmology Visual Science	Kallinikos, P	2004	209
Corneal Confocal Microscopy Detects Early Nerve Regeneration in Diabetic Neuropathy After Simultaneous Pancreas and Kidney Transplantation	Diabetes	Tavakoli, M	2013	200
Corneal confocal microscopy detects early nerve regeneration after pancreas transplantation in patients with type 1 diabetes	Diabetes Care	Mehra, S	2007	175
Small Nerve Fiber Quantification in the Diagnosis of Diabetic Sensorimotor Polyneuropathy: Comparing Corneal Confocal Microscopy With Intraepidermal Nerve Fiber Density	Diabetes Care	Chen, X	2015	172

**TABLE 6:** The top 10 most co-cited articles related to diabetic keratopathy.

Title	Journal	First Author	Year	Citations
Diabetic keratopathy	Transactions of the American Ophthalmological Society	Schultz R.O.	1981	110
Corneal structure and sensitivity in type 1 diabetes mellitus	Investigative Ophthalmology Visual Science	Rosenberg M.E.	2000	106
Corneal confocal microscopy: a non-invasive surrogate of nerve fibre damage and repair in diabetic patients	Diabetologia	Malik R.A.	2003	99
Surrogate markers of small fiber damage in human diabetic neuropathy	Diabetes	Quattrini C.	2007	94
Corneal confocal microscopy: a novel noninvasive test to diagnose and stratify the severity of human diabetic neuropathy	Diabetes Care	Tavakoli M.	2010	85
Differences in corneal thickness and corneal endothelium related to duration in diabetes	Eye	Lee J.S.	2006	74
Detection of diabetic sensorimotor polyneuropathy by corneal confocal microscopy in type 1 diabetes: a concurrent validity study	Diabetes Care	Ahmed A.	2012	70
Tear function and ocular surface changes in noninsulin-dependent diabetes mellitus	Ophthalmology	Dogru M.	2001	67
Advanced glycation end products in diabetic corneas	Investigative Ophthalmology Visual Science	Kaji Y.	2000	64
Diabetic complications in the cornea	Vision Research	Ljubimov A.V.	2017	64

### 3.7 Keywords

**FIGURE 7:**
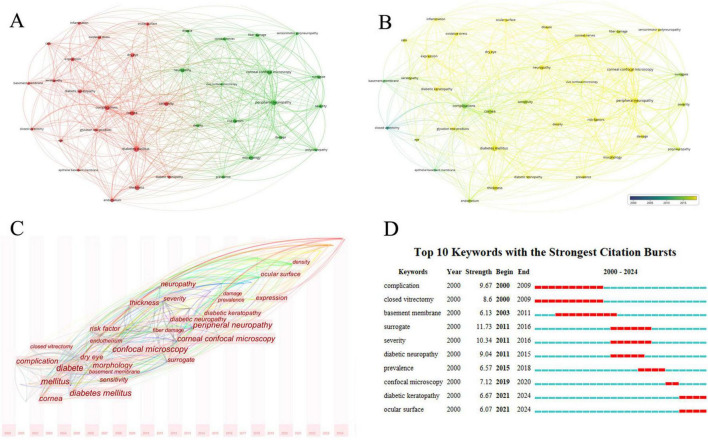
Analysis of keywords related to DK. **(A)** The co-occurrence network visualization map of keywords related to DK. The node size indicates the frequency of keywords, and different colors represent different co-occurrence clusters. **(B)** The co-occurrence network overlay visualization map of keywords related to DK. Blue represents the early research phase, and yellow represents the recent period. **(C)** The time zone view of keywords related to DK. The horizontal axis represents time, and the font size indicates the frequency of keywords. **(D)** The top 10 keywords with the strongest citation bursts related to DK from 2000 to 2024. The red segment of the blue line denotes the burst duration of a keyword.

Keywords reflect the main research content and direction of an article. Analyzing the high-frequency keywords in this field can help us understand the research focus and trends over time. Analysis of keywords with over 30 occurrences using VOSviewer revealed two major clusters: one around “complications”, “diabetes mellitus”, “cornea” and “sensitivity” and another around “corneal confocal microscopy” and “peripheral neuropathy” (Figure 7a). Keywords such as “complications”, “cornea” and “sensitivity” appeared earlier, while “corneal confocal microscopy” and “peripheral neuropathy” appeared later (Figure 7b). The time zone view of keywords analyzed by CiteSpace effectively reveals the changes in research directions. It can be observed that the keywords have evolved from “complications”, “diabetes mellitus” and “cornea”, to “corneal confocal microscopy” and “peripheral neuropathy”, and more recently to “ocular surface” (Figure 7c). The top burst words from 2000 to 2024 are shown in Figure 7d, with “surrogate” having the highest burst strength (11.73) from 2011 to 2016. Other keywords with high burst intensity mainly included “complications”, “sensitivity” and “diabetic neuropathy”.

## 4 Discussion

Past research on diabetic ocular complications primarily focused on diabetic retinopathy due to its severe impact on vision (10–12). However, DK is gaining increasing attention from researchers worldwide for two main reasons (13, 14). First, the prevalence of DK continues to increase with the increasing number of diabetes patients and the prolonged duration of the disease. Second, with the application of confocal microscopy, observing the morphology and quantity of corneal nerves has become an important surrogate marker for evaluating peripheral neuropathy in diabetic patients (15–17).

The increasing number of publications also confirms that DK is receiving more attention. The annual publication volume in this field shows a year-on-year increase, with explosive growth, especially after 2013. This surge is mainly due to the application of confocal microscopy, which allows for more intuitive observation of nerve damage in patients with DK. Additionally, damage to corneal nerve tissue serves as an important indicator for evaluating peripheral neuropathy in diabetic patients (17–19). The publication volume has not shown any decline in recent years, indicating that research in this field remains a hotspot.

Analyzing publications by country, institution, and author reveals the global distribution and characteristics of research in this field. Although the United States and China lead in terms of publication volume, their citation rates are not very high, and they share the same collaboration cluster. In contrast, England, Australia and Qatar have high citation rates, with the England-Australia-Qatar collaboration cluster being a key research focus. The University of Manchester in the UK, which ranks first in both the number of publications and citations, is especially noteworthy. The University of Queensland in Australia is also a research hub in this field for the country, ranking second in publication volume and having a high number of citations. Malik Rayaz from Qatar is the most prolific researcher in this field, making Qatar a leading research frontier, on par with traditionally developed countries such as the UK and Australia. Nathan Efron from Australia ranks second in publication volume and collaborates closely with Malik Rayaz. It is believed that core researchers like Malik Rayaz and Nathan Efron have made the UK-Australia-Qatar research collaboration circle the current global core in this field.

Research outcomes in this field are primarily published in ophthalmology and diabetes journals. Although more articles are published in ophthalmology journals, those in diabetology journals have higher impact factors and receive more citations. The main reason is that diabetes and its peripheral neuropathy constitute a larger research area, leading to a greater impact factor and more frequent citations for related articles (20, 21). Despite the lower impact factors of ophthalmology journals, those such as IOVS and Cornea are very influential within the ophthalmology field and merit attention from researchers (22, 23). Moreover, the research focus of articles published in ophthalmology journals differs from those in diabetes journals. Articles published in the diabetes journals primarily focus on corneal nerve damage detected by confocal microscopy, which serves as an indicator of diabetic peripheral neuropathy. In contrast, articles published in ophthalmology journals cover various aspects of diabetic keratopathy.

High-citation articles and main keyword analysis indicated that past research focused on observing diabetic corneal neuropathy and structural changes via cornea confocal microscopy. Studies have shown that diabetic patients have reduced corneal nerve fiber density, nerve branch density, and nerve fiber length (17, 18, 24). Damage to the corneal nerves in diabetic patients not only leads to decreased corneal sensitivity, making them more susceptible to external injuries, but also results in a reduction of neurotrophic substances in the corneal tissue (25–27). This lack of nutrients impairs the function of corneal stromal cells and epithelial cells. Additionally, corneal nerve fibers are crucial for maintaining healthy corneal epithelium and promoting wound healing after ocular trauma (28, 29). The loss of these nerve fibers leads to corneal epithelial damage and persistent nonhealing wounds. Moreover, confocal microscopy revealed a decrease in corneal epithelial cell density in diabetic patients, along with reduced activation and migration of these cells postinjury (30–32). Similarly, corneal stromal cells and endothelial cells in diabetic patients are also affected to varying degrees, showing a decrease in numbers (33, 34).

From the analysis of keyword clustering over time, time zone maps, and burst word analysis, we find that terms like “ocular surface”, “tear film” and “dry eye” have become hotspots in recent years. This shift indicates a broader interest beyond corneal pathology to the entire ocular surface, including the conjunctiva and tear film. Diabetic patients exhibit decreased tear film stability, increased osmolarity, and elevated levels of advanced glycation end products and inflammatory factors, all of which are detrimental to corneal epithelial repair (35–37). Immune cell activation, proteomic changes, and reduced mucin secretion in the conjunctiva also impact diabetic keratopathy to varying degrees (38, 39).

Keyword analysis also revealed that research on the mechanisms of DK and its associated neural damage is still limited. Previous studies have suggested that oxidative stress, neurotrophic deficits and inflammatory mechanisms play crucial roles in the pathogenesis of diabetic corneal neuropathy (40–42). Corneal epithelial damage is mainly related to the accumulation of advanced glycation end products on the ocular surface due to hyperglycemia (43, 44). Corneal stromal damage is linked to hyperglycemia-induced disruption of collagen subtypes and proteoglycans, affecting the structural integrity of the cornea (45, 46). These mechanisms are not prominent in the keyword network, suggesting that these areas have not yet become central to research in this field. Researchers need to further explore the key mechanisms underlying DK, or perhaps the pathogenesis of DK is multifactorial, lacking a single core mechanism.

## 5 Conclusion

This study systematically analyzed the literature on diabetic keratopathy published since 2000. We found that this field is gaining increasing attention and research interest. Visualization analysis provides a clear and rapid understanding of the current research status, collaboration networks, and research hotspots. Analyzing these hotspots suggests that future research may focus on the mechanisms of diabetic corneal neuropathy.

## Data Availability

The original contributions presented in the study are included in the article/supplementary material, further inquiries can be directed to the corresponding author.
